# Turnover of Focal Adhesions and Cancer Cell Migration

**DOI:** 10.1155/2012/310616

**Published:** 2012-01-26

**Authors:** Makoto Nagano, Daisuke Hoshino, Naohiko Koshikawa, Toshifumi Akizawa, Motoharu Seiki

**Affiliations:** ^1^Faculty of Pharmaceutical Sciences, Setsunan University, 45-1 Nagaotoge-cho, Hirakata, Osaka 573-0101, Japan; ^2^Division of Cancer Cell Research, Institute of Medical Science, University of Tokyo, 4-6-1 Shirokane-dai, Minato-ku, Tokyo 108-8639, Japan

## Abstract

Cells are usually surrounded by the extracellular matrix (ECM), and adhesion of the cells to the ECM is a key step in their migration through tissues. Integrins are important receptors for the ECM and form structures called focal adhesions (FAs). Formation and disassembly of FAs are regulated dynamically during cell migration. Adhesion to the ECM has been studied mainly using cells cultured on an ECM-coated substratum, where the rate of cell migration is determined by the turnover of FAs. However, the molecular events underlying the disassembly of FAs are less well understood. We have recently identified both a new regulator of this disassembly process and its interaction partners. Here, we summarize our understanding of FA disassembly by focusing on the proteins implicated in this process.

## 1. Introduction

Adhesion of cells to the ECM is key to the regulation of cellular morphology, migration, proliferation, survival, and differentiation [1, 2]. These functions are indispensable during development and for maintenance of tissue architecture and the induction of tissue repair. Integrins are the predominant receptors that mediate cell adhesion to components of the ECM [3–8]. Integrins are expressed on the cell surface as heterodimers comprised of noncovalently associated *α*- and *β*-subunits. Both subunits are type I transmembrane proteins containing both a large extracellular domain responsible for binding to ECM ligands and a cytoplasmic portion (CP) that recruits multiple intracellular proteins. Eighteen different *α*- and 8 *β*-subunits have been characterized in mammals, and 24 distinct integrin heterodimers have so far been identified [5, 9, 10]. Each integrin recognizes a distinct ECM ligand. As such, the repertoire of integrins expressed on the surface of a particular cell acts as a sensor of the ECM environment [5].

**Figure 1:**
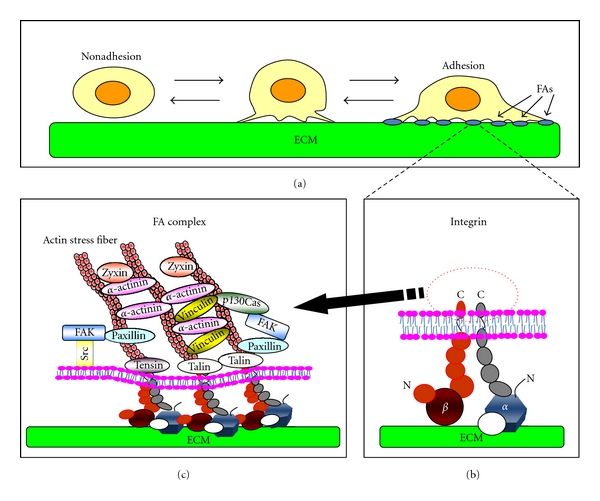
Integrin-mediated cell adhesion to the ECM. (a) Suspended cells adhere to the surface of ECM via integrins. Some of the nascent adhesion contacts grow and form mature focal adhesions (FAs). (b) Integrins function as a heterodimer composed of *α*- and *β*-chains. (c) The cytoplasmic portions of integrins recruit multiple cellular proteins and form cross-linked platforms to regulate both the actin cytoskeleton and signal transduction.

 Attachment of cells to ECM components induces clustering of integrins on the cell surface. The cytoplasmic portions of the clustered integrins then act as a platform for the recruitment of cellular proteins such as adaptor/scaffold and signaling proteins to the inner surface of the plasma membrane, where they form structures called focal adhesions (FAs) (Figure 1) [11–13]. The adaptor/scaffold proteins in FAs, such as talin, paxillin, tensin, p130Cas, and *α*-actinin, provide strong linkages to the actin cytoskeleton and, thereby, connect cells firmly to the ECM [14–18]. This linkage enables the generation of the tension necessary to alter cell morphology and the traction force necessary to move the cell body during migration. In addition, multiple signaling proteins, including kinases or phosphatases, are also recruited to FAs where they transmit ECM-derived signals to cellular pathways controlling proliferation, survival and migration [19–23]. In particular, two well-characterized tyrosine kinases, focal adhesion kinase (FAK) and Src, play central roles in integrin-mediated signaling cascades [20, 24, 25]. Since integrins have no intrinsic enzymatic activity, these tyrosine kinases transmit signals from FAs to the cellular machinery by phosphorylating multiple integrin-associated proteins [25–30]. Thus, both FAK and Src act as molecular switches that trigger a variety of cellular responses via FA complexes. There are many excellent reviews discussing how integrin-mediated signals regulate cellular behavior [20, 25, 31, 32].

The process of cell adhesion to the ECM has been studied by seeding cells onto an ECM-coated substratum in culture [33, 34]. These analyses contributed to the elucidation of the process of the initial attachment of cells to the ECM and the formation of integrin-mediated cell adhesion structures. However, cells must also detach from the ECM during migration, and the mechanism and regulation of the disassembly of cell adhesion structures is less well studied. In contrast to most review articles discussing cell adhesion, we focus here on our understanding of the turnover of FA complexes during cell migration.

## 2. Adhesion of Cells through the Formation of Focal Adhesion Structures

Cells adhere to the ECM via integrins and form FA complexes as discussed elsewhere [7]. Numerous proteins are involved in integrin-mediated cell adhesion, and these proteins are collectively referred to as the adhesome [35–38]. Among the latter, talin is a key regulator of the initial step of FA assembly [39–41]. Talin contains two unique domain structures, the head and rod domains [42–45]. The head domain mediates binding to the CP of the *β*-subunit of integrin, whereas the rod domain contains multiple binding sites for adhesome proteins, including one for the CP of *β*-integrin, two sites for actin, and multiple sites for vinculin. In addition, talin forms a dimer through its carboxy-terminal helix and thus serves as a core platform to expand intracellular structural frameworks mediated by protein-protein interactions. The binding of talin to integrin stabilizes the ligand-induced clustering of the latter at an initial step of FA formation by mediating crosslinking of integrins with filamentous actin (F-actin) and F-actin-binding proteins such as vinculin and *α*-actinin (Figure 3(a)) [14, 46–48]. This initial structure, called the nascent FA, is immature and often short lived [6]. However, some of the nascent FAs grow and form mature FAs that require actin-based tension regulated by the Rho small GTPase and its effector ROCK [6].

## 3. Regulation of Focal Adhesion Complexes during Cell Migration

**Figure 2:**
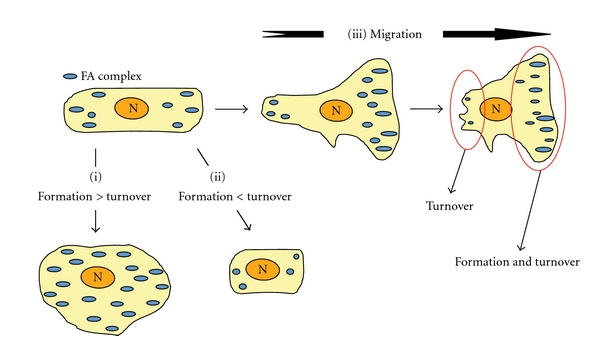
The formation and turnover of FAs during cell migration. The formation and turnover of FAs is crucial for cell adhesion to the ECM. A higher ratio of formation relative to turnover leads to stable adhesion (i). On the other hand, a higher ratio of turnover relative to formation leads to unstable adhesion (ii). During cell migration, both rapid formation and turnover of FAs are required at the leading edge of cell migration, whereas turnover of FAs is predominant at the rear (iii).

Stimulation of the formation of FA complexes enhances the adhesion of cells to the ECM, giving rise to cells with a spread morphology (Figure 2(i)). In contrast, destabilization of FAs reduces adhesion to the ECM and gives rise to spherical nonadherent cells (Figure 2(ii)). During cell migration on a substratum, FAs grasp the ECM so as to generate the forces necessary to pull the cell body forward. Subsequently, cells must release from the ECM, so as to continue cell movement. As such, directional migration of the cell requires continuous, coordinated formation and turnover of FAs at the leading edge of the cell body and release of this attachment at the rear (Figure 2(iii)) [49, 50]. Clustering of integrins is the initial step of cell adhesion and is stabilized to form FAs by linking to actin stress fibers in a process regulated by Rho/ROCK [6, 51, 52]. By contrast, extension of microtubules to FAs triggers their disassembly and induces the subsequent internalization of integrins from the cell surface [53–56]. Therefore, the assembly and disassembly of FAs are regulated by different mechanisms. Although the fate of the internalized integrins has not yet been established, several studies have reported the transport of internalized integrins from the rear to the leading edge of the cell body via intracellular vesicle trafficking [57–59]. This recycling of integrins may contribute to directional cell migration.

## 4. Factors Involved in the Disassembly of FAs

**Figure 3:**
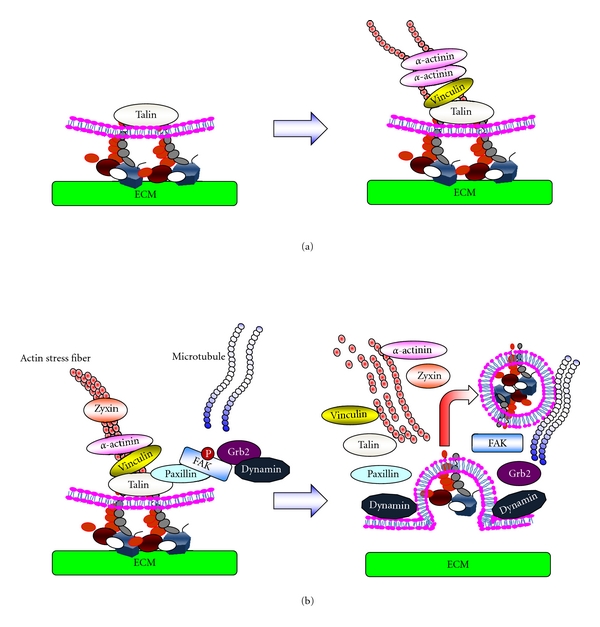
Formation and turnover of FAs. (a) The process of the formation of FAs. Attachment of cells to the ECM induces clustering of integrins at the attachment sites. Clustered integrins recruit cytoplasmic adaptor proteins such as talin to the cytoplasmic portion of the integrins. Actin-binding proteins such as vinculin and *α*-actinin then bind to talin and connect the ECM structure to the cytoskeleton via integrin. (b) The process of FA turnover. FAK phosphorylated at Tyr^397^ plays a role in recruiting the endocytosis regulator dynamin into FAs via interaction with the adaptor protein Grb2. The extension of MTs initiates the internalization of integrins in a dynamin-dependent manner. During the process of integrin endocytosis, rapid dephosphorylation of FAK at Tyr^397^ is observed.

The molecular events leading to FA disassembly are not yet well understood although some fragmentary knowledge has recently accumulated [54, 60, 61]. Most importantly, it has been established that microtubules (MTs) play a crucial role in inducing FA disassembly [54]. MTs extend to FAs and trigger the disassembly process. During the final stage, the internalization of integrins is mediated by dynamin, a GTPase that regulates endocytosis, and FAK is involved in the recruitment of dynamin into FAs (summarized in Figure 3(b)).

**Figure 4:**
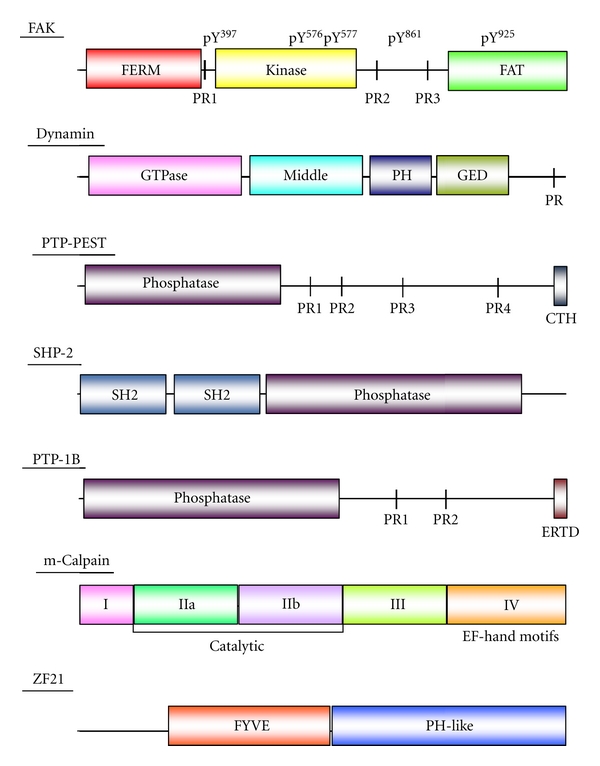
Domain structures of FA disassembly factors. FAK: FERM (protein 4.1, ezrin, radixin, and moesin homology), PR (proline-rich motif), FAT (focal adhesion targeting), pY (phosphorylated tyrosine), Dynamin: PH (pleckstrin homology), GED (GTPase effector domain), PTP-PEST: PR (proline-rich motif), SHP-2: SH2 (src homology 2 domain), PTP-1B: PR (proline-rich motif), ERTD (endoplasmic reticulum-targeting domain), m-Calpain: I (possible autoinhibitory region), IIa and IIb (protease domain), III (putative phospholipid-binding sites), and IV (the region containing 4 EF-hand motifs), ZF21: FYVE (Fab1, YOTB, Vac1, and EEA1), PH-like (pleckstrin homology-like).

 In the following sections, we summarize the proteins involved in disassembly and link their involvement in this process so as to generate a more coordinated model of disassembly based on recent findings. Various disassembly factors and their domain structures are schematically illustrated in Figure 4.

### 4.1. Microtubules

The importance of MTs for FA disassembly has been demonstrated using nocodazole, which disrupts polymerized MTs in cells adherent to the ECM [54, 56]. Exposure of cells to nocodazole stabilizes FA structures by preventing their disassembly and thereby enhances adhesion of cells to the ECM. The removal of the drug from the culture media initiates disassembly of FAs in a synchronous manner and recovery of MT structures [53, 54, 62]. Thus, the use of this drug allows us to analyze the FA disassembly process independently from FA formation. Tyrosine phosphorylation of proteins within FAs increases following exposure to nocodazole and decreases rapidly after its removal. Extension of MTs to FAs has been observed by live imaging microscopy, and targeting of MTs to FAs appears to trigger FA disassembly [54]. Since the MT motor protein, kinesin-1, has been implicated in regulating MT-induced FA disassembly [55], MTs may deliver disassembly factors to FAs in a kinesin-1-dependent fashion.

 As extension of MTs to FAs triggers release of cell adhesion and promotes cell migration, it is of interest how targeting of MTs to FAs is regulated during the induction of cell motility. Indeed, Rho family GTPases regulates the capture and stabilization of extended MTs to the cell cortex via their downstream effectors, and MTs in turn have been shown to affect the activity of Rho GTPases [63]. Although it is not precisely clear how MTs target FAs, actin filaments presumably play a role.

### 4.2. Kinesin-1

Kinesin-1 is a member of the kinesin superfamily of motor proteins and is also known as conventional kinesin [64, 65]. Kinesin-1 plays a crucial role in protein trafficking along polymerized MTs to the direction of plus end of latter. The inhibition of kinesin-1 in *Xenopus* fibroblasts, using either a specific antibody or forced expression of a dominant-negative mutant, leads to stabilization of FAs accompanied by an increase in their size and a reduction in their number, as was seen in cells exposed to nocodazole [55]. These findings suggest that kinesin-1 activity is necessary for the turnover of FAs. Although nocodazole inhibits the polymerization of MTs, inhibition of kinesin-1 activity affects neither the targeting of MTs to FAs nor the polymerization dynamics of MTs [55]. This suggests that FA disassembly factors are conveyed along MTs in a kinesin-1-dependent manner.

### 4.3. Focal Adhesion Kinase

FAK is involved in both maturation and turnover of FAs [20, 66]. However, FAK deficiency has a greater effect upon disassembly than upon formation of FAs, giving rise to a reduced rate of FA turnover leading to an increase in the level of steady-state FAs [60, 66]. FAK contains an N-terminal FERM (protein 4.1, ezrin, radixin, and moesin homology) domain, a central kinase domain, and a COOH-terminal focal adhesion-targeting (FAT) domain as illustrated in Figure 4. The FERM domain is found in many proteins and mediates protein-protein interactions [67, 68]. The FAK FERM domain has been shown to bind the CP of integrin *β*1 and growth factor receptors [69, 70]. Recent structure analysis of the FERM domain has indicated that it binds the catalytic cleft of the kinase domain [71]. This intramolecular interaction prevents autophosphorylation of Tyr^397^, which is a prerequisite for the successive phosphorylation of FAK by Src. Autophosphorylation of FAK at Tyr^397^ is elevated in highly motile and invasive cancer cells [72, 73]. Src binds to phosphorylated Tyr^397^ and further phosphorylates multiple tyrosine residues within FAK, including Tyr^576^ and Tyr^577^ within the kinase domain, Tyr^861^ located between the kinase and FAT domain, and Tyr^925^ within the FAT domain [20, 25]. Phosphorylation within the kinase domain is crucial for full kinase activity. Phosphorylated Tyr^861^ mediates the interaction of FAK with talin and paxillin [20, 25]. Phosphorylation at Tyr^925^ is necessary for the interaction of FAK with Grb2 [20, 25]. Binding of Grb2 to FAK helps recruit dynamin to FAs [54]. This ternary complex is responsible for the internalization of integrins and thereby induces turnover of FAs. However, the role of FAK during FA disassembly is not so simple. Whereas pTyr^397^ FAK is required for recruitment of dynamin, its dephosphorylation is induced after extension of MTs to FAs, and this is a prerequisite step for the successive disassembly of FAs [54, 62]. Thus, FAK is a central regulator of the formation and disassembly of FAs, and for the transmission of integrin-mediated signals. Nevertheless, FAK deficiency has little effect upon FA formation but has nevertheless been shown to stabilize FAs. The roles of FAK during FA formation might be performed by other redundant kinases or factors recruited to FAs.

### 4.4. Dynamin

Dynamin is a GTPase that was identified as an MT-binding protein [74]. Three independent dynamin genes have been identified. Dynamin I is expressed specifically in neurons, and Dynamin III is expressed exclusively in testis, lung, and brain, whereas Dynamin II is expressed ubiquitously [74]. The domain structure common to the dynamins is shown in Figure 4. Dynamin is required for the internalization of integrins during MT-dependent FA turnover [54]. The carboxyl terminus of dynamin contains a proline-rich (PR) motif, which is indispensable for assembly of a ternary complex with FAK and Grb2 [54]. The PR motif of dynamin also interacts with MTs. Dynamins recruited to the inner surface of the cells membrane assemble in a ring around FAs [54] and initiates the internalization of integrins when the FAs are sufficiently disassembled. FAK deficiency markedly reduces the accumulation of dynamin around FAs [54]. Interaction of the tubulin polymer with dynamin markedly increases the GTPase activity of the latter, although the physiological significance of this is unclear [75].

### 4.5. Phosphatases

A specific set of protein tyrosine phosphatases mediates dephosphorylation of FAK at Tyr^397^ after the extension of MTs to FAs [62]. These include PTP-PEST, SHP-2, and PTP-1B. However, it is not clear whether FA disassembly requires concerted action of all three phosphatases or whether the action of a single phosphatase is sufficient, depending on the cellular context.

PTP-PEST is known to regulate cell adhesion and migration (Figure 4) [76]. As Zheng et al. have reported, PTP-PEST dephosphorylates FAK at Tyr^397^ upon activation by an oncogenic Ras-induced signal [77, 78]. Ras induces the activation of ERK via the Fgd1-Cdc42-PAK1-MEK1 cascade ultimately resulting in interaction between FAK and PTP-PEST. Activated ERK phosphorylates FAK at Ser^910^, and the phosphorylated Ser^910^ and the adjacent Pro^911^ residue serves as a binding site for peptidyl-prolyl *cis/trans* isomerase (PIN1). PIN1 stimulates the binding of FAK to PTP-PEST, in a fashion dependent upon the isomerase activity of PIN1, although the exact role of the isomerase activity is not clear. PTP-PEST then dephosphorylates pTyr^397^ [79]. Intriguingly, substitution of FAK Tyr^397^ by Phe promotes metastasis of v-H-Ras-transformed rat fibroblasts.

SHP-2 can also dephosphorylate FAK at Tyr^397^ [80]. SHP-2 contains two SH2 domains at its N-terminus (Figure 4), and the N-terminal most of the two acts as an intramolecular inhibitor of the phosphatase activity [81]. This inhibition can be released by Gab2, a pleckstrin homology (PH) domain-containing docking protein. Gab2 binds the N-terminal SH2 domain and exposes the phosphatase domain of SHP-2 by releasing the intramolecular inhibition [81]. Deficiency of SHP-2 in cultured cells increases the number of FAs and impairs cell migration [82]. These findings are reminiscent of the phenotype of FAK-deficient cells. However, there is no clear evidence that SHP-2 localizes to FAs during their turnover. SHP-2 might be recruited to FAs by interacting with the phosphorylated tyrosines of Gab2 via its two SH2 domains.

 There are several substrates for PTP-1B in FAs, including FAK, Src, and *α*-actinin [83, 84]. PTP-1B is a complicated regulator of FAK. It directly mediates dephosphorylation of pTyr^397^ [84] but can also promote phosphorylation of the same tyrosine residue by Src [83]. As Zhang reported, *α*-actinin plays a key role in the dual functions of PTP-1B [84]. *α*-Actinin phosphorylated at Tyr^12^ promotes dissociation of Src bound to FAK at pTyr^397^. This allows PTP-1B to dephosphorylate the exposed pTyr^397^. On the other hand, PTP-1B can dephosphorylate *α*-actinin pTyr^12^ so as to increase the *α*-actinin-free Src pool that is then available to phosphorylate FAK. At the same time, PTP-1B can activate Src by dephosphorylating Src pTyr^527^, which mediates intramolecular inhibition of Src activity. Overall, dephosphorylation of FAK by PTP-1B enhances subsequent phosphorylation of FAK by Src. These functions of PTP-1B may play roles in the dynamic turnover of FAs during dynamic cell attachment rather than simply by promoting detachment.

### 4.6. m-Calpain

m-Calpain, also known as Calpain-2, is a member of the calpain family of intracellular calcium-dependent proteases [85]. It comprises five functionally and structurally distinct domains. Domain I is a possible autoinhibitory region, and it is cleaved off by autolysis. Domain II is a catalytic domain composed of two split subdomains (IIa and IIb) linked by a loop called the catalytic cleft. Domain III is a putative regulatory region of the protease activity, and it contains phospholipid-binding sites. Domain IV contains four EF-hand motifs that are necessary for binding calcium.

 m-Calpain has been shown to regulate the turnover of FAs by cleaving multiple FA-related proteins such as talin, FAK, and paxillin [61, 86–88]. Talin is a well-established substrate of m-Calpain during the turnover of FAs [61, 89]. m-Calpain cleaves a site between the head and the rod domains and thereby triggers structural breakdown of the FA framework [61]. FAK is also cleaved by m-Calpain between the two C-terminal PR domains [87]. Breakdown of FAs by m-Calpain also requires MTs [90]. Even though the precise role of MTs in the breakdown of FAs by m-Calpain is unclear, ZF21 presumably plays a role as explained in next section [62, 91]. FAK can bind both ERK/MAPK and m-Calpain, and it might be a platform where m-Calpain can be activated by the ERK/MAPK [92]. Cleavage of components of FAs by m-Calpain presumably facilitates internalization of integrins by disrupting interconnected large structure of FAs. 

### 4.7. ZF21

ZF21 contains a FYVE domain, which binds to phosphatidylinositol-3-phosphate that is enriched in the lipid layers of plasma membranes. Although there are 38 FYVE domain-containing proteins in mammals, they do not necessarily have common domain structures or functions [93]. ZF21 initially attracted our attention as a possible interaction partner of the cytoplasmic tail of the membrane type metalloproteinase, MT1-MMP, but it was later determined to be a regulator of FA turnover [62]. ZF21 is expressed almost ubiquitously in various types of adhesive cells. The FYVE domain of ZF21 is located in the middle in the protein, and the C-terminal region of the protein contains a novel protein fold that is similar to the PH domain but is lacking the positively charged amino acids necessary to bind phospholipid [94]. Interestingly, ZF21 binds multiple FA disassembly proteins, including FAK, *β*-tubulin, m-Calpain, and SHP-2 [62, 91, 94]. The FYVE domain of ZF21 binds FAK, and the PH-like domain binds *β*-tubulin. Almost the entire ZF21 polypeptide chain is required for binding m-Calpain and SHP-2. Substitution of the FYVE domain of ZF21 with a corresponding domain derived from EEA1, another member of the FYVE domain-containing proteins, abolishes its ability to bind FAK and abrogates its ability to mediate MT-induced FA disassembly [94].

**Figure 5:**
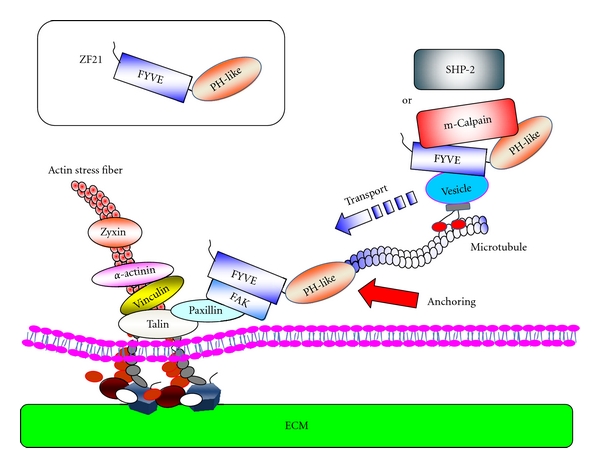
A model for the recruitment of disassembly factors to FAs. In this model, ZF21 conveys FA disassembly factors via intracellular vesicle transport on MTs. ZF21 associates with endosomal vesicles by binding to phosphatidylinositol-(3)-phosphate via its FYVE domain. m-Calpain and SHP-2 can be loaded onto ZF21 carried by the vesicles. ZF21 also can be found in FAs by interacting with FAK, and the ability of ZF21 to bind *β*-tubulin may act as a docking function for the extended MTs into FAs in order to unload the conveyed factors at the destination.

 Knockdown of ZF21 expression in cells prevents MT-induced FA disassembly, as well as disassembly-related events, such as dephosphorylation of FAK at pTyr^397^ and internalization of integrins [62]. Binding of ZF21 to FAK is important for the regulation of FA disassembly because substitution of the FYVE domain with that of EEA1 abolishes both FAK binding and FA disassembly [94]. The PH-like domain is also indispensable for the activity of ZF21 [94]. Taken together, these findings suggest that ZF21 associates with endosomal vesicles moving on MTs via an interaction between the FYVE domain and phosphatidylinositol-3-phosphate within the vesicle membrane. The PH-like domain, which mediates an interaction with *β*-tubulin, may help stabilize the interaction of ZF21 with MTs and then ride on vesicles. The ability of ZF21 to bind SHP-2 and m-Calpain may facilitate the transport of the latter to FAs via vesicles loaded onto MTs (Figure 5). Upon targeting of MTs to FAs, ZF21 may be transferred to FAs since it can bind FAK, and the ZF21 transferred to the FAs may subsequently anchor the MTs to the FAs. Gab2 in FAs may facilitate the dephosphorylation of FAK by SHP-2 carried in on the MTs. These events are presumably followed by breakdown of FA components by the proteolytic activity of m-Calpain.

 Importance of ZF21 for cell migration gave us a clue to understand its role in FA turnover [62]. Knockdown of ZF21 expression by shRNA in cancer cells induced cell spreading on the ECM and suppressed cell migration. Integrin-mediated cell adhesion and migration are important during cancer cell invasion and metastasis although FA-like structures are not obviously recognizable in most cells surrounded by ECM. Indeed, knockdown of ZF21 expression in human mammary carcinoma MDA-MB231 cells suppresses metastatic colony formation in the lung following injection of the cells into the tail vein of mice [94]. However, it is possible that ZF21 regulates metastasis of cancer cells by mechanisms distinct from the regulation of the turnover of FAs.

## 5. Conclusion

Our understanding of the mechanism of FA turnover remains fragmentary. However, the mechanisms governing the migration of cells due to regulated adhesion are crucial to the understanding of cancer cell invasion and metastasis. Turnover of FAs is initiated by the extension of MTs to FAs and is completed by the internalization of integrins from the cell surface. Several factors have been implicated in the process of FA disassembly. In particular, the recently identified ZF21 has shed light on this process, owing to its ability to bind multiple proteins involved in FA disassembly. It is of note that FAs are not observed in cells cultured in a collagen lattice, indicating that the presence of integrin-based cell adhesion structures is dependent upon whether the cells are adhering to a rigid surface (2D) or are embedded within a 3-dimensional ECM (3D) [95, 96]. Advanced imaging technologies are powerful tools to elucidate the dynamic roles of FA disassembly factors during cell migration and invasion.
